# Patient-specific gait pattern in individuals with patellofemoral instability reduces knee joint loads

**DOI:** 10.1038/s41598-024-79021-x

**Published:** 2024-11-18

**Authors:** Bernhard Guggenberger, Brian Horsak, Andreas Habersack, Annika Kruse, Colin R. Smith, Hans Kainz, Martin Svehlik

**Affiliations:** 1 Department of Orthopaedics and Trauma, Medical University of Graz, Graz, Austria; 2 Neuromechanics Research Group, Department of Biomechanics, Kinesiology and Computer Science in Sport, Centre for Sport Science and University Sports, University of Vienna, Auf der Schmelz 6a (USZ II), 1150 Vienna, Austria; 3 Institute of Physiotherapy, FH JOANNEUM University of Applied Sciences, Graz, Austria; 4 Center for Digital Health and Social Innovation, St. Pölten University of Applied Sciences, St. Pölten, Austria; 5 Institute of Human Movement Science, Sport and Health, University of Graz, Graz, Austria; 6 Department of Biomedical Engineering, Steadman Philippon Research Institute, Vail, CO USA

**Keywords:** Paediatric research, Biomedical engineering

## Abstract

Patellofemoral instability is influenced by morphological factors and associated with compensational alterations in gait pattern. Recent simulation studies investigated the impact of knee morphology on the stability and loading of the patellofemoral joint but neglected the patient-specific gait pattern. The aim of this study was to investigate the impact of patient-specific gait pattern on muscle forces and joint loading in individuals with patellofemoral instability. Musculoskeletal simulations with a model including a twelve degrees of freedom knee joint were performed based on three-dimensional motion capture data of 21 individuals with chronic patellofemoral instability and 17 healthy control participants. The patellofemoral instability group walked with a less flexed knee joint and reduced knee flexion and abduction moments compared to the control group, which required less quadriceps muscle forces. Lower quadriceps muscle forces resulted in a reduction of tibiofemoral and patellofemoral joint contact forces despite similar walking velocities between both groups. Furthermore, we observed decreased lateralizing patella forces in subjects with patella instability, which could potentially reduce the risk of patella dislocation. Our findings highlight the importance of accounting for the patient-specific gait pattern when analysing knee loads in individuals with patellofemoral instability.

**Supplementary Information:**

The online version contains supplementary material available at 10.1038/s41598-024-79021-x.

## Introduction

Patellofemoral instability (PFI) is a common knee condition in children and adolescents, which describes inadequate guidance of the patella in the trochlea groove^1^. It has been shown that ligament properties, bony structure, axial alignment between femur and tibia, as well as neuromuscular factors influence the stability of the patellofemoral joint^1–3^. A lack of stability can further lead to patella dislocations^4^. Patella dislocations are associated with functional decline and pain^3^. Untreated PFI and dislocations can result in limitations in physically strenuous activity and cartilage degradation^5^. Moreover, PFI has been identified as a potential risk factor for patellofemoral arthritis^6^. Patella dislocations often occur during physical activity, early knee flexion (up to 30 degrees), external rotation of the tibia and contraction of the quadriceps^7,8^. As these factors arise in gait during the early single stance phase, individuals with PFI alter their gait pattern compared to healthy people^9^. However, subject-specific gait pattern and its impact on the knee joint loading in patients with PFI have not been investigated yet. A better insight in how the walking pattern of individuals with PFI affect knee joint loads, could help to improve and tailor treatments.

Clinical three-dimensional gait analysis is a well-established method to quantitatively and objectively describe and analyze the gait pattern from a kinematic (joint angles) and kinetic (joint moments) perspective^10^. Recent studies used this approach to investigate compensational gait strategies in individuals with PFI^9,11,12^. These studies showed, that individuals with PFI walk with less hip and knee flexion during the entire gait cycle^11,13^. Further, their internal knee flexion moments during stance were decreased^11^. In the frontal plane, increased hip adduction angles and abduction moments as well as an increased genu valgum posture was observed in individuals with PFI^13,14^.

In addition to standard clinical three-dimensional gait analysis, musculoskeletal simulations can be used to increase our insights in the biomechanical pathomechanism of PFI^15^. Musculoskeletal modelling enables the estimation of internal joint forces and cartilage loading, which cannot be measured in a non-invasive way^16,17^. Recent research used this approach to investigate the impact of different bone geometries on the patellofemoral stability and loading^18^. A simulation study showed that during squatting cartilage stress at the patellofemoral joint is sensitive to external rotations of the femur^19^. Several recent studies used statistical shape models combined with musculoskeletal simulations based on the walking pattern of healthy participants to investigate the influence of patellofemoral geometry on patellofemoral biomechanics^18,20–22^. They found that the geometry can alter patella kinematics (orientation and translation) and patellofemoral loading^18^. Furthermore, they showed that in knees with a shallow trochlea groove a medial or lateral displacement of the tibial tubercle can lead to a decreased patella stability^21^ and greater differences in patella position and cartilage contact pressure^20^. One recent study further found a lower ratio of quadriceps tendon forces to patella ligament forces in Wiberg type III patellas compared to more symmetrical patellas^22^. Although all the above-mentioned studies increased our insights in patellofemoral biomechanics, a major limitation of these studies is that they mainly used data of healthy individuals to drive their musculoskeletal simulations thereby neglecting patient-specific gait patterns^18,19,21,22^.

Although it is known that individuals with PFI walk with gait deviations^9,11,12^, little is known about the impact of the patient-specific walking strategies on tibiofemoral and patellofemoral joint loading. A better understanding of altered joint loads in these individuals could support the development of more targeted PFI treatment strategies. To close this gap, we investigated the impact of patient-specific gait patterns in individuals with PFI on the knee joint loading. We hypothesized that individuals with PFI alter their gait pattern to reduce the forces acting on the patella and thus try to reduce the risk of patella luxation.

## Methods

We retrospectively analysed three-dimensional motion capture data from a clinical database. Musculoskeletal simulations were performed to investigate how the gait pattern, knee joint loading and patellofemoral contact pressure differ between individuals with PFI and a control group of healthy participants. The ethics committee of the Medical University of Graz (IRB00002556, 34-181 ex 21/22) approved this study. Due to the retrospective nature of this study the requirement of informed consent was waived by the ethics committee of the Medical University of Graz. All methods were performed following relevant regulations and guidelines.

### Participants

For the PFI group, inclusion criteria were an age between 10 and 18 years and unilateral recurrent PFI, which was defined as at least three patella dislocations. Subjects with other lower leg injuries or neurological diseases were excluded. Between 2010 and 2020, 256 individuals with PFI were treated at our paediatric orthopaedic centre. Forty-five out of 256 patients experienced three or more patella dislocations and were referred to gait analysis. Twenty-four of these 45 patients were excluded due to bilateral involvement (n = 16), other knee injuries (n = 3), neurological disease (n = 1) or poor data quality (n = 4). Hence, three-dimensional gait analysis data, i.e. marker trajectories and ground reaction forces, of 21 individuals with unilateral recurrent PFI, were included in this study.

As control group, 17 typically developing adolescents, were included. Inclusion criteria for the control group were an age between 10 and 18 years and no injuries at the lower extremities. We ensured that sex and age distribution in the control group matched the participants of the PFI group.

### Three-dimensional motion capturing

All gait analysis data were captured in the same laboratory. All participants walked barefoot at self-selected walking speed. Gait analysis data were collected using a ten-camera infrared-based motion capture system (Vicon Motion Systems, Oxford, UK) operated at a sampling rate of 120 Hz. The gait data for the PFI group were collected using the plug-in gait marker set^23,24^ and for the control group using the modified Cleveland marker set^25^. Hip joint centres were calculated for all participants based on a modified version of the Harrington equation^26^, using the pelvis width as input^27^. Four force plates operated at a sampling rate of 1080 Hz (Advanced Mechanical Technology Inc., Watertown, MA, USA) captured ground reaction forces synchronously to the kinematic trajectories. Force plate threshold was set to 15 N vertical force and gait events were detected automatically for each trial. Trials with no full foot contact solely on one force plate were excluded.

### Musculoskeletal simulation

We scaled a generic OpenSim model developed by Lenhart et al.^28^ to the anthropometry of each participant using the three dimensional motion capturing data^29^. The same anatomical markers were used to scale the model to the anthropometry of each individuum. The model included 44 muscle–tendon actuators encompassing hip, knee and ankle joint muscles, 14 ligament bundles designed as nonlinear springs, a six degrees of freedom tibiofemoral and patellofemoral joint as well as cartilage surfaces for the tibiofemoral and the patellofemoral joint^28^. The additional degrees of freedom at the knee joint facilitate the computation of secondary knee kinematics, including tibiofemoral abduction and rotation, as well as patellofemoral translation and rotation along the three anatomical axes. Due to a limited number of markers on the foot, the metatarsophalangeal joint and the subtalar joint were locked in each model. Ankle joint plantar-/dorsiflexion remained unlocked in all models. The maximum isometric muscle forces were scaled according to the squared body height^30^.

**Fig. 1:**
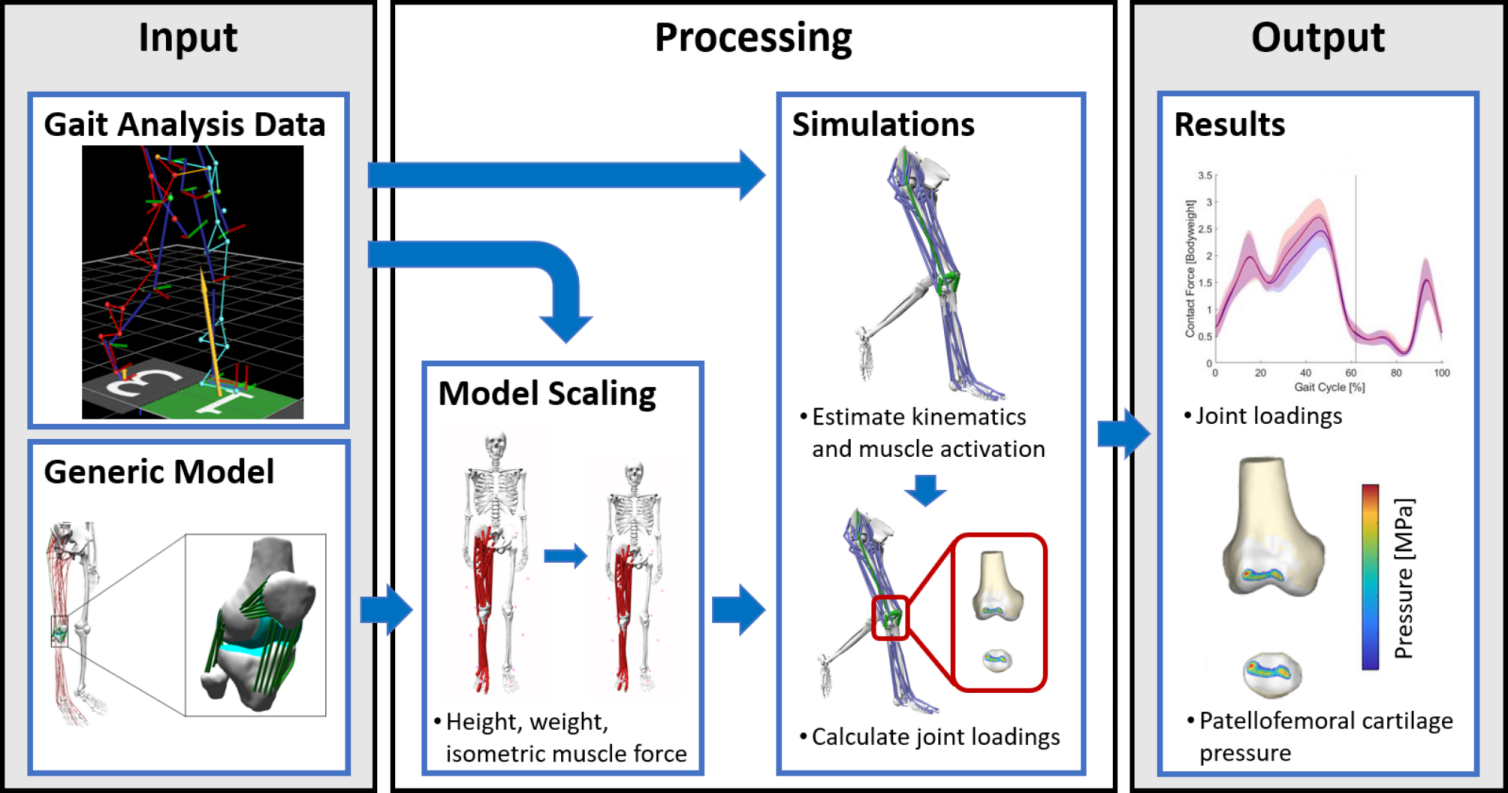
Schematic representation of the simulation workflow. Gait analysis data and a generic model served as input data. The generic model was scaled in height, weight and maximum isometric muscle force for each subject. Concurrent optimisation of muscle activations and kinematics routine was used to estimate joint kinematics, joint kinetics and muscle forces. Further joint contact forces and patella cartilage pressure were calculated.

We calculated joint angles, external joint moments, muscle forces, joint contact forces and patellofemoral contact pressures. Musculoskeletal simulations were performed based on each participant’s scaled model and the corresponding gait data (Fig. 1). Primary kinematics, such as pelvis motion, hip flexion, hip abduction, hip internal/external rotation, knee flexion, and ankle flexion, were calculated by minimizing the weighted sum of squared differences between the experimental and model marker positions. Additionally, external joint moments were computed using inverse dynamics. The concurrent optimisation of muscle activations and kinematics (COMAK) routine^31^ was used to concurrently solve the model for muscle activation and secondary knee kinematics. This routine concurrently resolves secondary knee kinematics by minimizing the weighted sum of squared muscle activations and contact energy. Utilizing these outcomes, joint reaction loads were subsequently calculated^32^. Cartilage contact pressures were calculated using a non-linear elastic foundation model, predicated on the penetration depth between overlapping cartilage surface meshes^33–35^. A uniform cartilage thickness of 3 mm on each surface (6 mm total thickness at the tibiofemoral and patellofemoral joint) was assumed^28,36^. Elastic modulus and Poisson-ratio for the cartilage were set to 5 MPa and 0.45, respectively^37,38^. Maximum marker errors for the inverse kinematic calculations were below OpenSim’s best practice recommendations for all participants^39^.

### Data analysis

We time normalized each gait cycle to 101 time points (0–100% gait cycle). Ground reaction forces, external moments, contact forces and contact pressure were normalized to the body weight (BW = body mass × g) of each participant. At least five gait cycles were processed and averaged for each participant. Discrete parameters were calculated in prior to averaging curves and separately averaged. Statistical analysis for discrete parameters was performed using SPSS v27 (IBM, New York, USA) and Matlab (The Mathworks Inc., Natick, MA, USA). To statistically compare the waveforms between the PFI and control group we used Statistical parametric mapping^40^, i.e. SPM1D package for Matlab (http://www.spm1d.org).

Gait kinematic and knee joint loading waveforms in the sagittal, frontal and transversal planes as well as estimated rectus femoris, vastus medialis and vastus lateralis muscle force waveforms were compared between the PFI and control group. As patella dislocations happen in a comparable pattern as at the beginning of single supported stance phase (knee flexion up to 30 degrees and contraction of the quadriceps), we additionally investigated the maximum tibiofemoral and patellofemoral joint forces as well as the patellofemoral cartilage pressure at that time point.

To compare discrete parameters such as demographic data, spatiotemporal gait parameters, and the maximum knee joint contact forces and patellofemoral contact pressure at the beginning of the single supported stance phase we used the following procedure. Initially a Shapiro–Wilk test was used to check for normal distribution per goup^41^. If data was normally distributed we used independent t-tests for comparison, otherwise Mann–Whitney U tests^42,43^. We calculated Cohen’s d to estimate the effect size^44^.

To compare the ground reaction force, joint kinematics, external moments, knee joint contact forces and contact pressure waveforms between individuals with PFI and the control group we checked for normal distribution of the waveforms using the normality test provided within the SPM1D package. For normally distributed data we used the parametric version of the two-tailed t-test, for non-parametric data the nonparametric version. Alpha level was set to 0.05. To address for multiple testing we used Bonferroni corrections on different parameter levels as follows^45^. For the comparison of patellofemoral cartilage pressure waveforms between both groups resulting p-values were correct for three family-wise comparisons (peak pressure, mean pressure, pressure area). Further we corrected the comparisons for the tibiofemoral and patellofemoral joint contact force waveforms separately for three family-wise comparisons (vertical, anterior–posterior and medio-lateral forces). For the maximum value of joint contact forces and maximum values of patellofemoral cartilage pressure in the first 30 percent of stance phase resulting p-values were correct in accordance with the corresponding waveforms for three family-wise comparisons (vertical, anterior–posterior, medio-lateral tibiofemoral force, vertical, anterior–posterior, medio-lateral patellofemoral force, peak pressure, mean pressure, and pressure area).

## Results

The PFI group and control group showed no significant differences in age, sex, height, body weight and body-mass-index (Table 1). Radiographic measurements were available for 20 subjects of the PFI group. The PFI group presented an average Caton-Deschamps index of 1.20 ± 0.14, distance between the tibial tuberosity and the trochlea groove of 15.6 ± 3.1 mm, femoral anteversion of 24.5 ± 9.7 degrees, and tibial torsion of 34.6 ± 8.0 degrees. In regards to trochlear dysplasia, the participants were classified according to the Dejour classification as follows: A (n = 7), B (n = 5), C (n = 6), and D (n = 2). Cadence, stride length, step width and gait speed normalized to the leg length did not significantly differ between both groups. Compared to the control group, the PFI group walked with longer stance phase and shorter swing phase. Double supported stance phases, the loading response and the pre-swing phase were significantly longer and the single stance phase shorter in the PFI group compared to the control group.

**Table 1:** Demographic information for the patellofemoral instability group and the control group.

	PFI group	Control group	p-Value	Cohen’s d
Age [years]	15.6 (1.8)	15.2 (1.2)	0.370	0.28
Sex [male/female]	3/18	3/14		
Height [m]	1.6 (0.1)	1.7 (0.1)	0.529	0.21
Body Weight [kg]	61.1 (11.9)	60.7 (11.2)	0.905	0.04
Body-Mass-Index	21.4 (3.9)	21.7 (3.3)	0.867	0.06
Cadence [1/min]	112.1 (9.9)	111.1 (5.8)	0.734	0.11
Stride length [m]	127.2 (8.8)	128.7 (11.1)	0.651	0.15
Step width [cm]	8.3 (1.8)	8.9 (1.4)	0.208	0.42
Gait speed normalized to leg length [1/s]	0.15 (0.01)	0.15 (0.02)	0.800	0.08
**Stance phase [%]**	**62.5 (1.4)**	**60.7 (1.4)**	** < 0.001**	**1.29**
**Swing phase [%]**	**37.5 (1.4)**	**39.3 (1.4)**	** < 0.001**	**1.29**
**Loading Response [%]**	**11.7 (1.5)**	**9.9 (1.3)**	** < 0.001**	**1.28**
**Single stance phase [%]**	**37.8 (1.6)**	**40.2 (1.5)**	** < 0.001**	**1.55**
**Pre swing phase [%]**	**13.0 (1.5)**	**10.6 (1.5)**	** < 0.001**	**1.56**

Parameters are presented as follows: mean (standard deviation). Parameters with significant differences between groups are highlighted bold. All parameters were normally distributed and therefore analysed by independent t-test.

PFI, patellofemoral instability.

### Ground reaction forces

Mediolateral ground reaction forces were significantly lower in individuals with PFI compared to healthy controls, whereas vertical and anterior–posterior ground reaction forces did not differ between both groups (Supplementary information 1).

### Kinematics

**Fig. 2:**
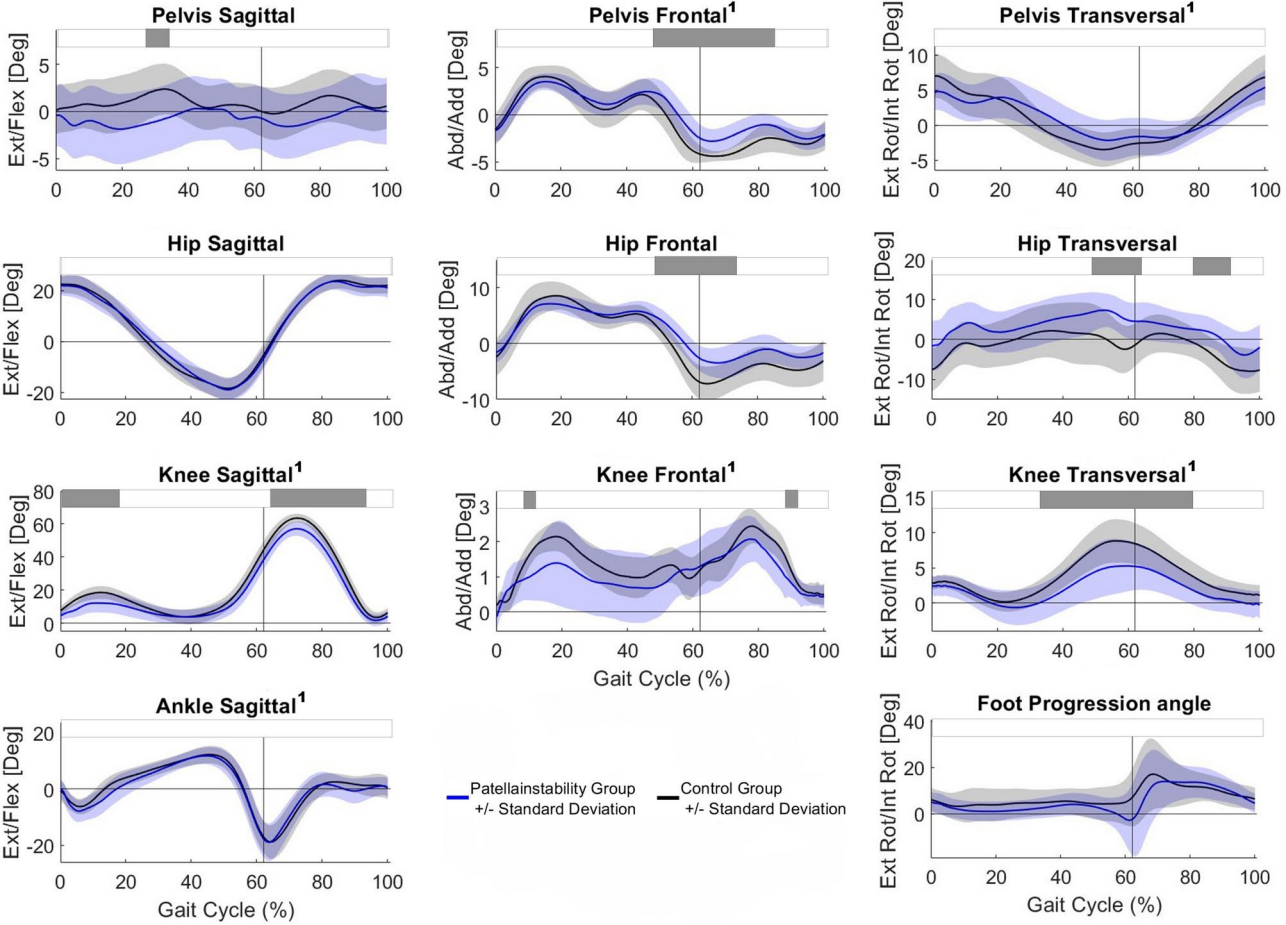
Kinematics. The blue and black line represent the mean and one SD for the PFI and the control group, respectively. Horizontal grey bars at the top represent significant differences identified via statistical parametric mapping (SPM) between both groups. A superscript 1 beside the title symbols non-parametric waveform distribution and therefore use of non-parametric version of SPM.

The PFI group walked with lower knee flexion angles during loading response and swing phase. Further, the PFI group walked with lower pelvis and hip abduction at the end of the stance phase, an increased hip internal rotation and knee external rotation (Fig. 2).

### Kinetics

**Fig. 3:**
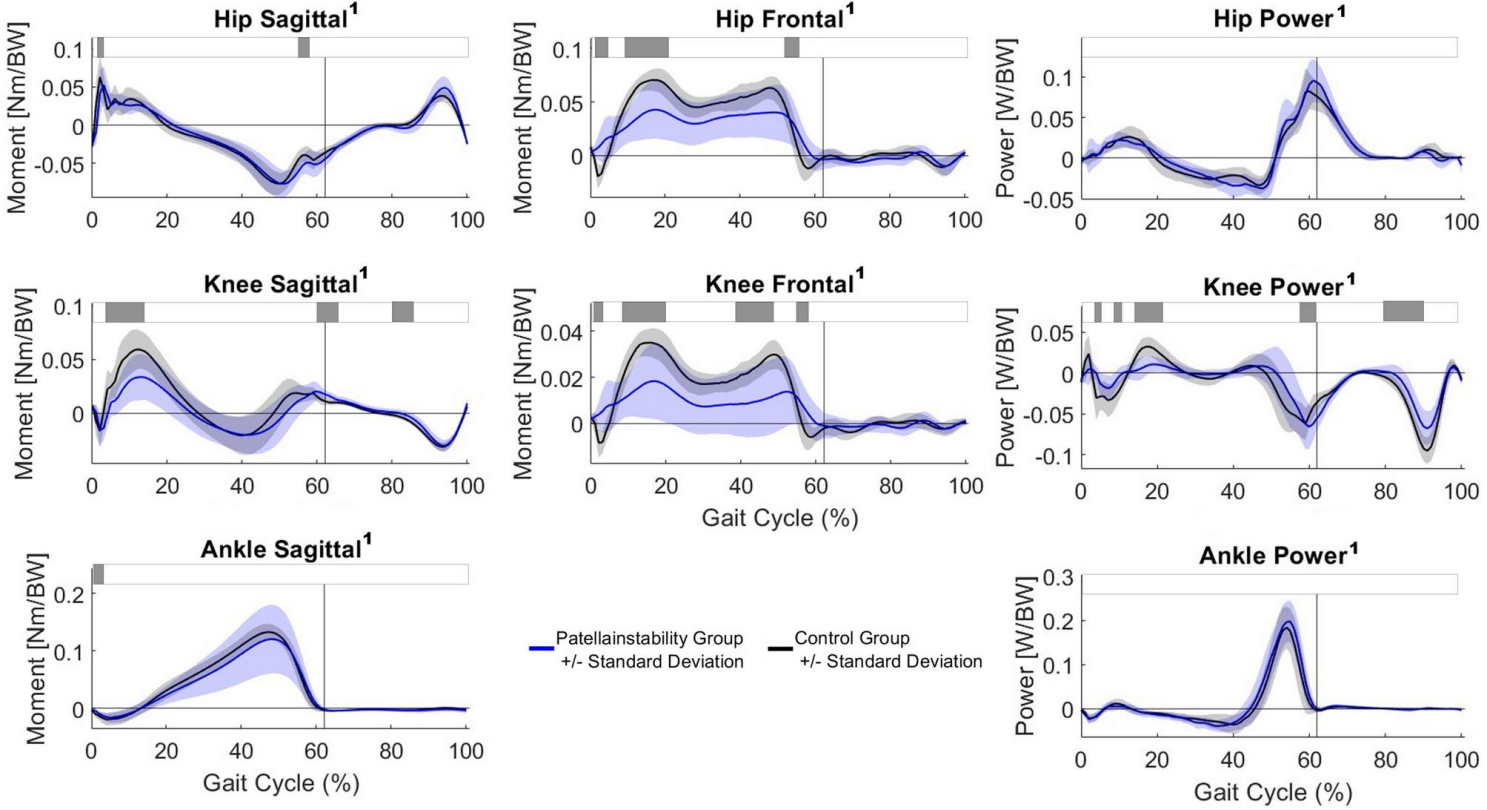
Kinetics. The blue and black line represent the mean and one SD for the PFI and the control group, respectively. Horizontal grey bars at the top represent significant differences identified via statistical parametric mapping (SPM) between both groups. A superscript 1 beside the title symbols non-parametric waveform distribution and therefore use of non-parametric version of SPM. BW, body weight.

The PFI group walked with a significant lower knee flexion moment at loading response compared to the control group. In the frontal plane, the PFI group displayed lower hip abduction and internal rotation moment and lower knee abduction moment compared to the control group. Frontal plane moments showed a larger standard deviation during the stance phase in the individuals with PFI (Fig. 3). The PFI group showed decreased joint power in loading response and midstance, as well as in late swing phase.

### Muscle forces

**Fig. 4:**
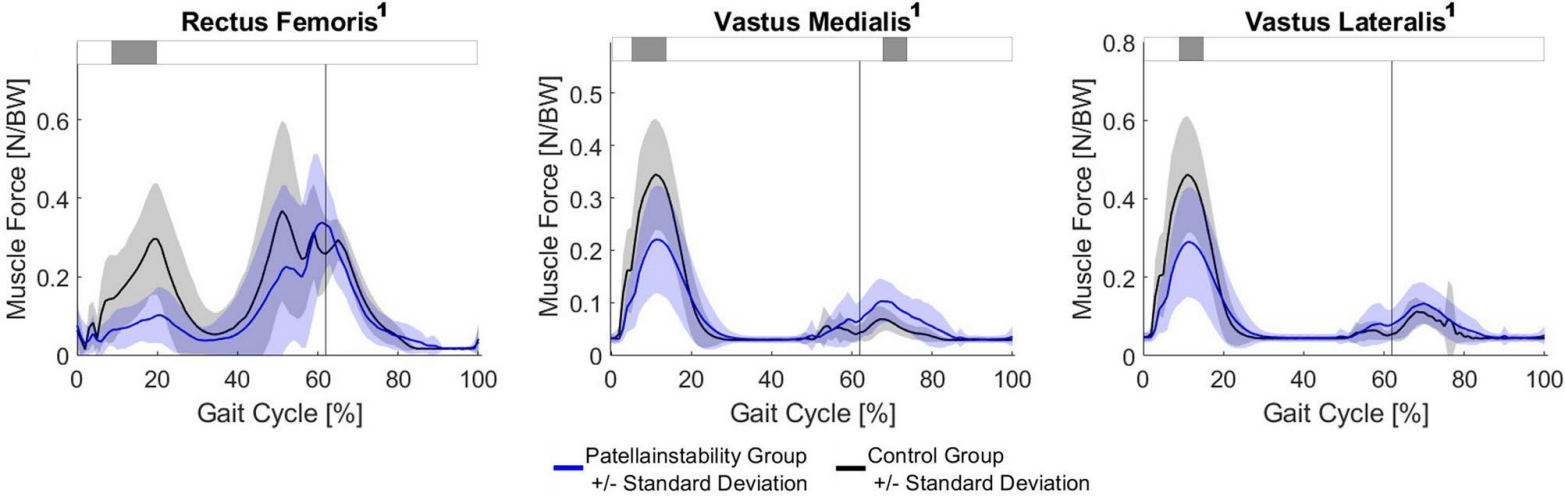
Muscle forces. The blue and black line represent the mean and one SD for the PFI and the control group, respectively. Horizontal grey bars at the top represent significant differences identified via statistical parametric mapping (SPM) between both groups. A superscript 1 beside the title symbols non-parametric waveform distribution and therefore use of non-parametric version of SPM. BW, body weight.

The PFI group walked with significant lower rectus femoris, vastus medialis and vastus lateralis muscle force in loading response (Fig. 4). Further, they walked with more vastus medialis muscle force in early swing phase.

### Joint contact forces and patella cartilage pressure

**Fig. 5:**
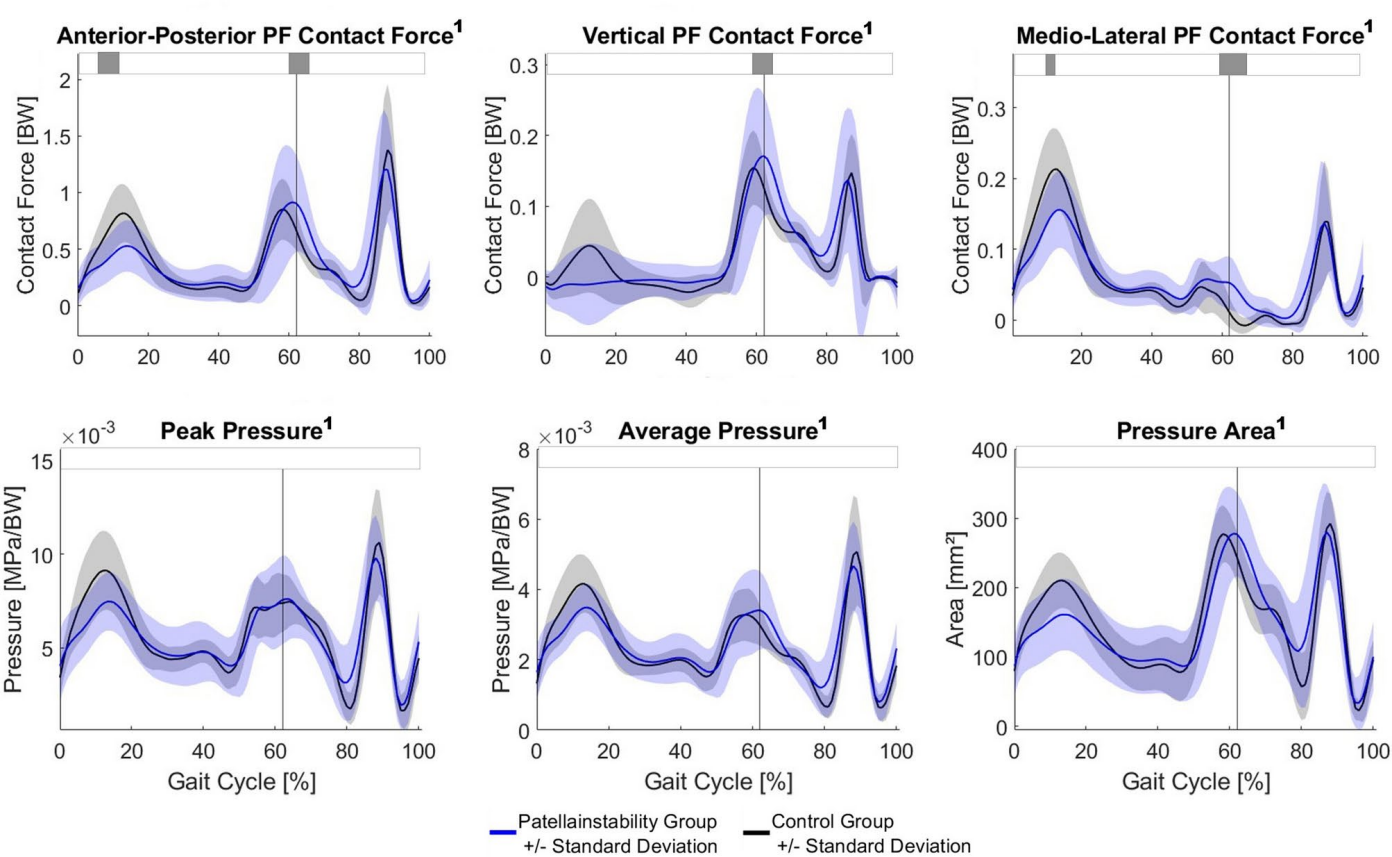
Patellofemoral joint contact forces as well as patellofemoral cartilage pressure and contact area. The blue and black line represent the mean and one standard deviation for the PFI and the control group, respectively. Horizontal grey bars at the top represent significant differences identified via statistical parametric mapping (SPM) between both groups. A superscript 1 beside the title symbols non-parametric waveform distribution and therefore use of non-parametric version of SPM. BW, body weight; PF, patellofemoral.

The PFI group showed lower tibiofemoral and patellofemoral joint contact forces in the loading response phase (Fig. 5 and Supplementary information 2). Additionally, the PFI group walked with higher patellofemoral joint contact forces at the beginning of swing phase. Maximum and mean patellofemoral cartilage pressures as well as contact area during the loading response phase tends to be lower in the PFI compared to the control group (no significant difference) (Fig. 5).

### Maximum contact forces and maximum patellofemoral contact pressure

The PFI group showed lower maximum forces and maximum pressures, as well as a lower maximum contact area, compared to the control group during the first 30 percent of the stance phase (loading response phase and mid stance phase) (Table 2).

**Table 2:** Maximum forces and pressure within the first 30 percent of the gait cycle.

	PFI group	Control group	p-value	Cohen’s d
**Vertical Tibiofemoral F [N/kg]**	**2.49 (0.43)**	**3.01 (0.55)**	**0.009**	**1.06**
**A-P Tibiofemoral F [N/kg]**	**0.33 (0.13)**	**0.46 (0.13)**	**0.012**	**1.01**
M-L Tibiofemoral F^1^ [N/kg]	0.13 (0.07)	0.17 (0.05)	0.054	0.66
Vertical Patellofemoral F^1^ [N/kg]	0.01 (0.04)	0.05 (0.06)	0.063	0.83
**A-P patellofemoral F [N/kg]**	**0.55 (0.23)**	**0.84 (0.26)**	**0.003**	**1.19**
**M-L patellofemoral F**^**1**^** [N/kg]**	**0.16 (0.05)**	**0.22 (0.06)**	**0.012**	**1.02**
**Peak pressure [MPa/kg]**	**0.08 (0.01)**	**0.097 (0.02)**	**0.030**	**0.91**
**Mean pressure [MPa/kg]**	**0.0036 (0.0006)**	**0.0043 (0.0008)**	**0.027**	**0.89**
**Pressure area [mm**^**2**^**]**	**164.66 (51.17)**	**212.13 (39.71)**	**0.009**	**1.02**

Parameters are presented as follows mean (standard deviation). Parameters with significant differences between groups are highlighted bold.  Parameters with a superscript 1 were not normally distributed and therefore analysed using the Mann–Whitney U test.

A-P, anterior–posterior; F, force; M-L, medio-lateral; PFI, patellofemoral instability.

## Discussion

The aim of this study was to investigate the impact of altered walking patterns in adolescents with PFI on knee joint loading. Individuals with PFI displayed differences in their gait pattern compared to a typically developing control group. More specifically, individuals with PFI presented altered gait kinematics and kinetics that resulted in a reduction of the quadriceps muscle force in loading response. Unloading of the quadriceps muscle led to a reduction of the tibiofemoral and patellofemoral joint contact forces. In agreement with our hypothesis the patient-specific gait pattern reduced medial–lateral PF contact forces during the first 30 percent of the gait cycle. Interestingly, even though our simulations estimated lower maximum patellofemoral cartilage pressure during the first 30 percent of gait cycle in the PFI group, we found no significant differences between groups for this parameter in the waveforms.

The PFI group walked with a longer stance phase, longer double supported stance phase and shorter single stance phase compared to the control group. An increase in double support can provide more stability, as it allows to adjust gait and correct deviations^46^. This supports the hypothesis that individuals with PFI alter their gait due to a feeling of instability and use the double supported phases to support the affected leg as long as possible. Another reason, especially for prolongation of loading response phase, might be the longer time for load shifting between legs. In loading response excentric knee flexion is used for shock absorbing^47^. Longer loading response duration could allow to reduce the use of the knee joint flexion for damping and therefore lower knee joint angles. This consideration is supported by the observed significantly lower knee power in loading response phase in our individuals with PFI compared to the control group (Fig. 3).

The PFI group walked with less knee flexion during loading response compared to the control group. A previous study showed a relationship between reduced knee flexion and decreased dorsiflexion (i.e. plantarflexion/knee extension couple) in individuals with PFI^11^. This relationship is often called the plantarflexion/knee-extension coupling and is thought to increase the stability of the knee joint, despite lower quadriceps muscle activity, by pulling back the tibia via soleus muscle activity^48^. In contrast to previous findings, we found no significant difference in ankle kinematics in the PFI group compared to the control group^11^.

The PFI group walked with internal hip rotation and external rotation of the tibia compared to the control group. This is a pattern frequently seen in individuals with PFI^9^. Further, it is in coherence with the increased femoral anteversion (24.5° ± 9.7°) and tibial torsion (34.6° ± 8.0°) in our PFI group. External tibia rotation is a predisposing factor for patella dislocation^8^. Furthermore, internal rotation of the femur affects the Q-angle and thus lateralize the direction of rectus femoris muscle force^49^. Therefore, deviation in hip and knee rotation might be part of the primary pathology and not a secondary compensation of gait^50^. Future research, focussing on gait retraining, should investigate the impact of treating gait pattern in the transversal plane on the stability of the patellofemoral joint^51,52^.

The PFI group walked with reduced external knee flexion moments during the loading response phase compared to the control group. The knee flexion angle alters the lever arm between the knee joint and ground reaction force^53^. Hence, the less flexed knee might be partially responsible for the reduced knee flexion moment. Lower knee moments reduce the necessity of high knee extensor muscle forces and therefore lead to an unloading of the knee joint.

In the frontal plane, the PFI group walked with reduced hip and knee joint moments. This might be caused by compensatory trunk kinematics which alters the direction of ground reaction force and thus reduce the frontal plane moments^54^. Though not included in the scope of this project, future research could focus on the impact of trunk movement in gait pattern of individuals with patellofemoral instability. The observed combination of differences in joint kinematics and medio-lateral ground reaction force might explain the reduced knee and hip abduction moments.

Musculoskeletal simulations showed that gluteus maximus compensates a lack of gluteus medius strength to generate hip abduction moment^55^. Additionally to the hip abduction moment, the gluteus maximus produces a hip extension moment, which is compensated by the rectus femoris and leads to an increased co-contraction and therefore joint loads at the hip and knee joint^56^. Individuals with PFI and a weak gluteus medius^57^ could avoid this mechanism with an altered gait pattern which reduces the hip abduction moment. Thus, the observed reduction in hip abduction moments could be a compensatory mechanism of individuals with PFI, to unload the knee joint. Contrary to our findings, one study found higher abduction knee moments in individuals with PFI^12^.

We observed a reduction of the tibiofemoral and patellofemoral joint contact forces in loading response in our participants with PFI compared to the control group. The PFI group walked with altered gait kinematics and kinetics (i.e. reduced knee flexion angle and moment, reduced hip abduction moment), which led to reduced quadriceps muscle forces. Walking with a less flexed knee joint to reduce quadriceps muscle force is called quadriceps avoidance pattern^11^ and is also known from other pathologies, e.g. people with patellofemoral pain syndrom^58^. This quadriceps avoidance pattern reduces the pull of the quadriceps muscle on the patella and consequently decreases the compressional force acting on the knee joint^59^.

Differences in joint kinematics and kinetics in our subjects with PFI lowered lateral patellofemoral contact force. A lateralizing force, especially in combination with a disadvantageous geometry (e.g. a shallow trochlea) increases the probability of a patella dislocation^60^. Besides that, increased femoral anteversion and hip internal rotation, can lead to an increased lateralizing force due to altered muscle lines of action of the rectus femoris and the vastus lateralis muscles^49^. A mechanism relevant to individuals with PFI, as higher femoral anteversion is common in those subjects^61^ and was also observed in our cohort.

Another reason for the quadriceps avoidance gait pattern could be pain. At six months after the last dislocation, a study showed load-dependent pain in individuals with PFI (39% of patients suffered from pain while running, minimal pain was reported while sedentary activities)^62^. Since the impact and load during walking is somewhere between running and sedentary activity, we expect little pain in our PFI group. A prospective study showing similar gait pattern in individuals with PFI compared to our PFI group reported no pain in their cohort and thus no relation between pain and walking strategy^11^. Future research should focus on a comprehensive pain screening to evaluate the relationship between gait pattern and pain for PFI in detail.

Patellofemoral cartilage pressure waveforms showed no significant differences in pressures in the PFI compared to the control group. The PFI group walked with a reduced knee flexion in loading response and thus reduced contact area between patella and trochlea groove^63^. The calculation of contact pressure depends on the applied force and the area. Thus, a reduction of the contact area might explain why we did not find significant differences in cartilage pressure in the waveforms despite the lower joint contact force. In contrast, the maximum pressure values during the first 30 percent of gait cycle showed significant lower pressures in the PFI group, and thus showed a reduced patella cartilage loading in individuals with PFI.

This study has some limitations. Firstly, we have no comprehensive information about perceived pain, exact number of dislocations and subjective assessment of knee stability. Therefore, we are limited in our interpretation and we do not know if gait pattern and knee contact forces were influenced by these factors. It is possible that some of the subjects suffered from pain and therefore altered their gait pattern to unload the knee joint^3^. A prospective study, however did not find a relationship between pain and gait pattern in individuals with PFI^11^. Second, metatarsal and subtalar joints were locked in all models, which could have slightly altered joint kinematics and joint loading in our simulations. This is a common procedure in musculoskeletal simulations, when only two markers are available at the foot segment^64–66^. Another limitation of the present study is, that the gait analysis data of the PFI group and the control group were collected with two different marker sets. This limitation is inherent to the retrospective nature of this study. However, a previous study demonstrated that the marker model has negligible impact on the simulation results^67^, and we therefore do not expect a relevant impact on our findings. Further, PFI is highly influenced by the bony geometry, e.g. shape of the trochlea groove^1–3^. Deviations in joint geometry can lead to changes in the contact area^20^. As our musculoskeletal models were all based on the same bony geometry our estimated pressures might not represent the real pressures. Our study, however enabled us to quantify differences in knee loads and patellofemoral cartilage pressure solely caused by the patient-specific gait pattern. Future research based on medical-imaging informed models could focus on the combined impact of patient-specific gait pattern and bone geometry on knee joint loads in people with PFI.

## Conclusion

This study showed how individuals with PFI alter their gait pattern to reduce knee joint loads. Compared to healthy controls, our individuals with PFI walked with less knee flexion during the stance phase and therefore required less quadriceps muscle forces. Lower quadriceps muscle forces resulted in a reduction of tibiofemoral and patellofemoral joint forces. Especially the decreased lateralizing force on the patella could potentially decrease the risk of patella dislocation and might explain why people with PFI walk with an altered gait pattern. Our findings highlight the importance of accounting for the patient-specific gait pattern when analysing knee loads in people with PFI.

## Electronic supplementary material

Below is the link to the electronic supplementary material.

Supplementary Material 1

Supplementary Material 2

## Data Availability

The datasets used and analysed during the current study are available from the corresponding author on request.
