# Supplementary material for: Patient-specific gait pattern in individuals with patellofemoral instability reduces knee joint loads
Source: Sci Rep. 2024 Nov 18;14:28520. doi: 10.1038/s41598-024-79021-x (PMC11574134; doi:10.1038/s41598-024-79021-x)
Supplement: Supplementary file 2 — Supplementary Material 2 [file 41598_2024_79021_MOESM2_ESM.docx]

**Supplementary information 2:**


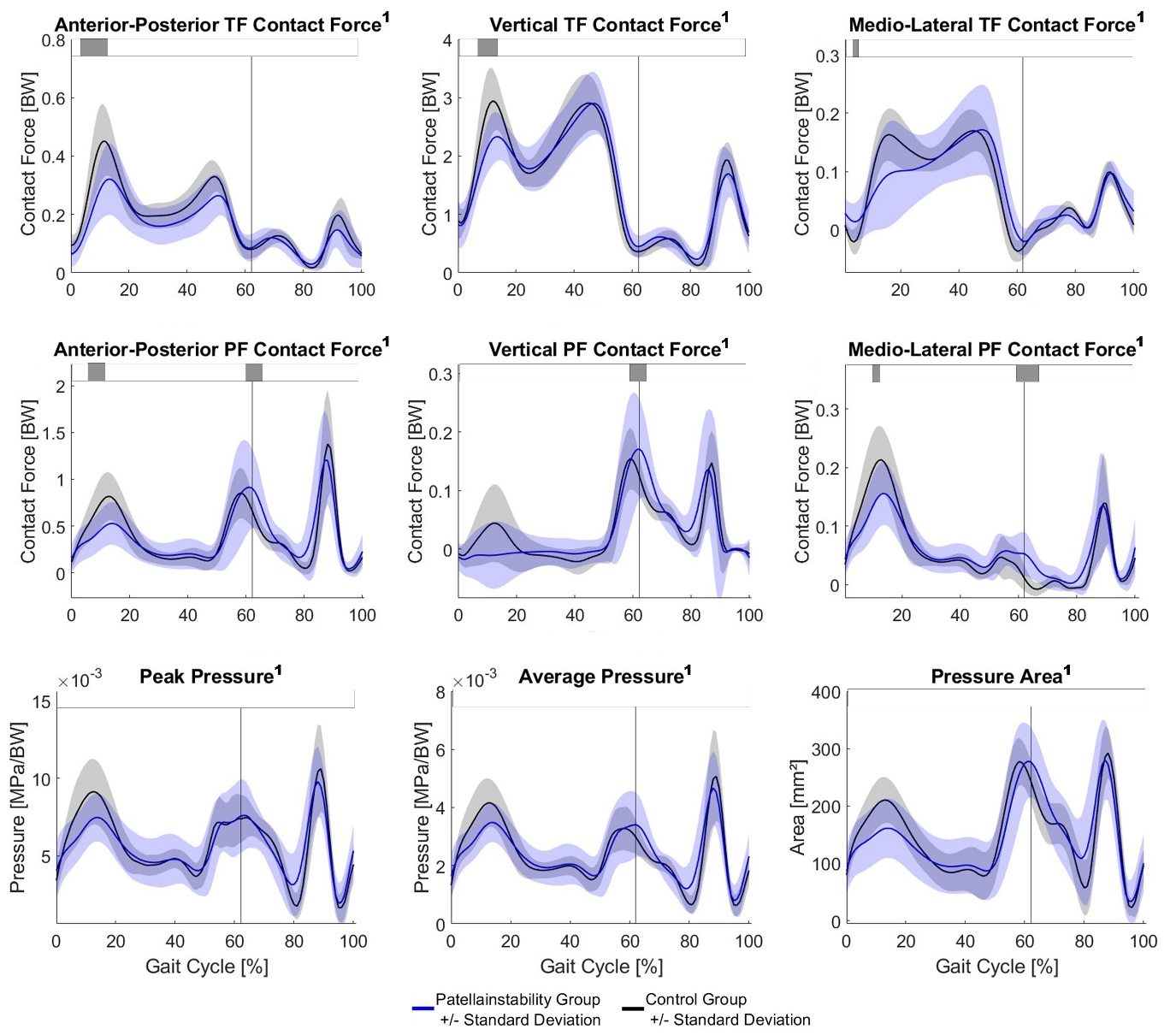

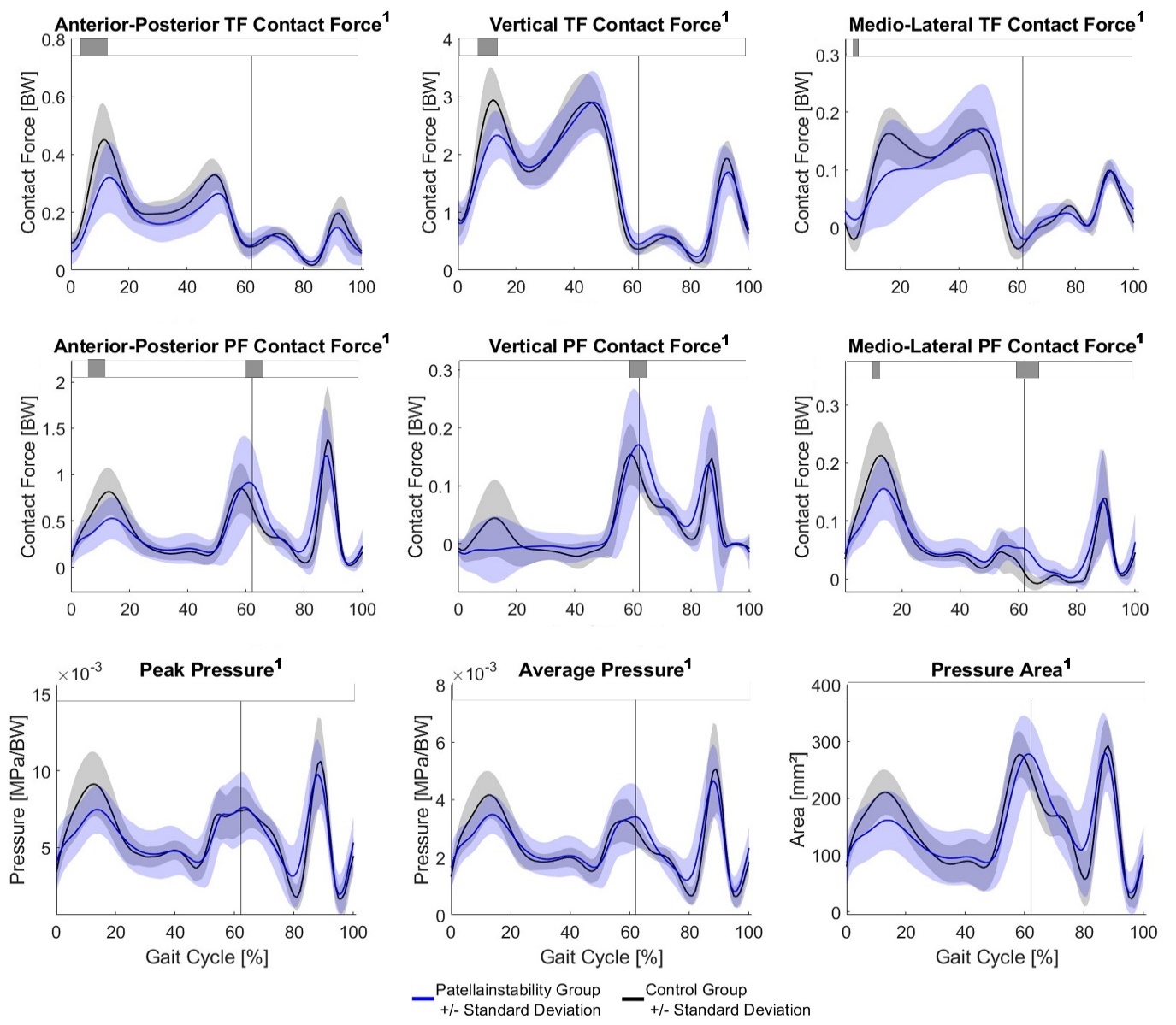


Tibiofemoral joint contact forces. The blue and black line represent the mean and one standard deviation for the PFI and the control group, respectively. Horizontal grey bars at the top represent significant differences identified via statistical parametric mapping (SPM) between both groups. A superscript 1 beside the title symbols non-parametric waveform distribution and therefore use of non-parametric version of SPM. BW = body weight, TF = tibiofemoral
